# Supplementary material for: Inflammatory factors and risk of lung adenocarcinoma: a Mendelian randomization study mediated by blood metabolites
Source: Front Endocrinol (Lausanne). 2024 Aug 27;15:1446863. doi: 10.3389/fendo.2024.1446863 (PMC11384989; doi:10.3389/fendo.2024.1446863)
Supplement: Supplementary file 1 [file DataSheet1.zip › supplementary files/S2/GCST90200134/sensitivity-analysis.pdf]

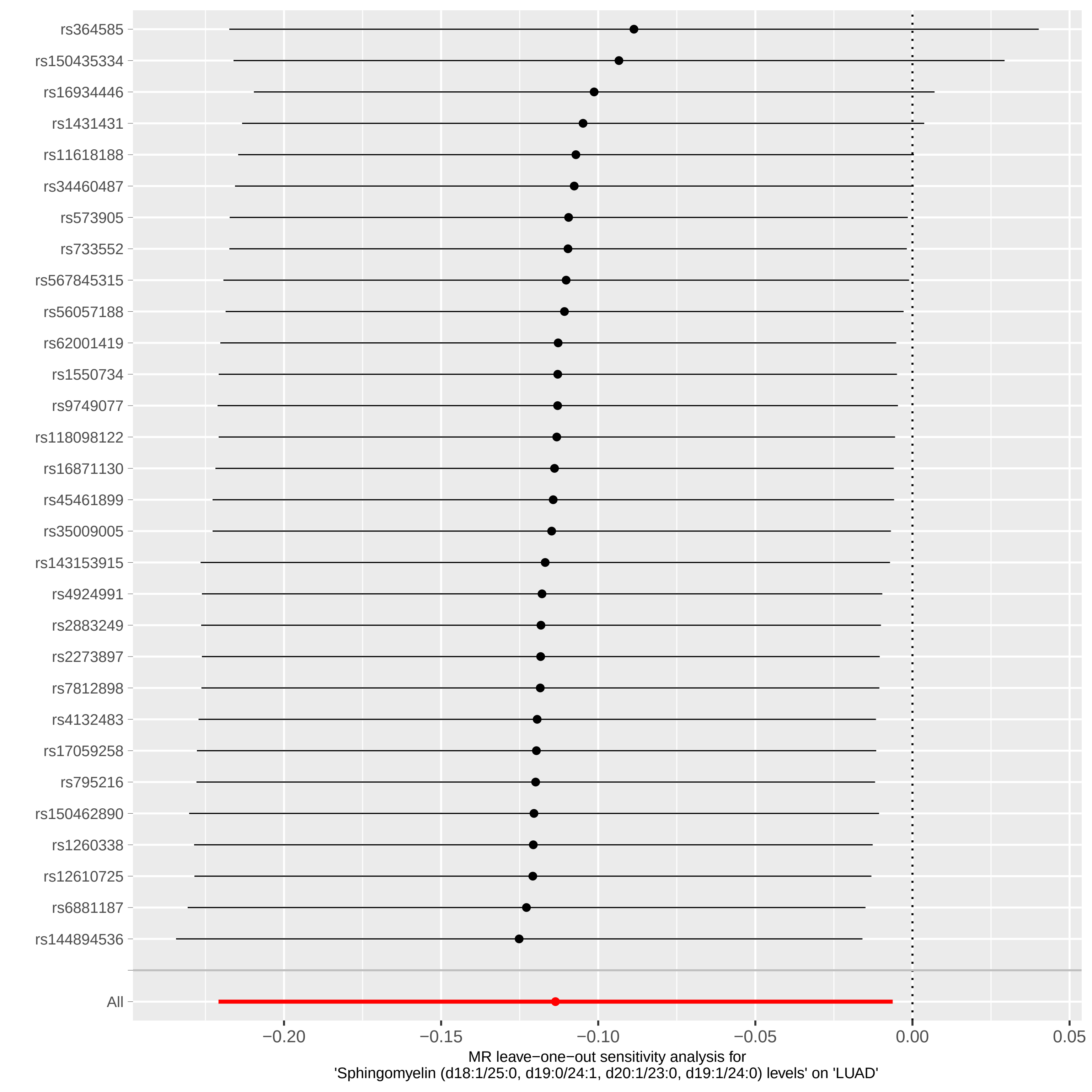
rs364585
rs150435334
rs16934446
rs1431431
rs11618188
rs34460487
rs573905
rs733552
rs567845315
rs56057188
rs62001419
rs1550734
rs9749077
rs118098122
rs16871130
rs45461899
rs35009005
rs143153915
rs4924991
rs2883249
rs2273897
rs7812898
rs4132483
rs17059258
rs795216
rs150462890
rs1260338
rs12610725
rs6881187
rs144894536
All
−0.20
−0.15
−0.10
−0.05
0.00
0.05
MR leave−one−out sensitivity analysis for
'Sphingomyelin (d18:1/25:0, d19:0/24:1, d20:1/23:0, d19:1/24:0) levels' on 'LUAD'
